# Optimization of Tribological Properties of Shot-Peened Surfaces via Multi-Criteria Decision-Making Using TOPSIS and GRA

**DOI:** 10.3390/ma18163733

**Published:** 2025-08-09

**Authors:** Andrzej Dzierwa, Izabela Miturska-Barańska

**Affiliations:** 1 Faculty of Mechanical Engineering and Aeronautics, Rzeszow University of Technology, Powstancow Warszawy 8 Street, 35-959 Rzeszow, Poland; 2 Faculty of Mechanical Engineering, Lublin University of Technology, Nadbystrzycka 36 Street, 20-618 Lublin, Poland; i.miturska@pollub.pl

**Keywords:** grey relational analysis, technique for order preference by similarity to ideal solution, friction, wear, surface topography, shot peening

## Abstract

The article presents a comparative analysis of experimental results from tribological tests conducted using a ball-on-disc system, applying two multi-criteria decision-making methods: Grey Relational Analysis (GRA) and TOPSIS (Technique for Order Preference by Similarity to Ideal Solution). The aim of the study was to identify the most advantageous combinations of input parameters—load, sliding speed, and sliding distance—while simultaneously evaluating three output criteria: volumetric wear (VD), coefficient of friction (CoF), and weight loss (WL). The analysis covered 27 test variants, with different weighting factors assigned to each criterion to reflect their practical significance (0.35 for VD, 0.45 for CoF, and 0.2 for WL). The results obtained using the GRA method showed good agreement with the TOPSIS rankings in identifying the best-performing variants, although differences were observed due to the distinct algorithms used to evaluate trade-offs. The optimal solutions were characterized by low wear, a low coefficient of friction, and minimal weight loss. The study demonstrates the effectiveness of both methods for tribological analysis and suggests that their combined use can serve as a robust tool for optimizing the operating conditions of friction nodes.

## 1. Introduction

The long-term and reliable performance of machine elements is largely influenced by the quality of the surface layer formed during manufacturing processes. The functional properties of these elements can be modified and improved through various surface modification methods [1]. One such method is shot peening, which offers several significant benefits, particularly for components subjected to material fatigue, abrasion, stress corrosion, or intensive cyclic loading. Shot peening is classified as a mechanical surface treatment that involves bombarding the material’s surface with small balls—made of steel, ceramic, glass, or plastic—at high velocities [2]. The impacts cause localized plastic deformation and compress the material at the point of contact, generating a layer of compressive residual stresses that inhibit crack propagation. In addition, shot peening can positively affect the microstructure of the surface layer, leading to significant improvements in hardness and wear resistance [3].

The authors of [4] reported a significant influence of shot peening on the fatigue strength of materials, attributed to changes in surface properties such as grain size, hardness, and residual stress. Their study on a 6082 T6 aluminum alloy used various shot materials including silica, alumina, aluminum, and zinc. Silica and zinc shots extended the fatigue life of the alloy, while alumina and aluminum shots had a detrimental effect. Better fatigue performance was associated with shots that induced higher compressive stresses. Raghuvaran et al. [5] also observed a positive impact of shot peening on fatigue strength. Testing on Al7075-SiC composites showed an increase in fatigue strength from 156.5 MPa to 174 MPa, indicating potential for improved performance in practical applications. Similarly, the authors of [6] reported a notable improvement in the fatigue strength of compacted graphite iron (CGI), with bending fatigue strength increasing by 8% for CGI with casting coating and by 9% for machined surfaces. Wu et al. [7] subjected an Al-7Si-0.3Mg alloy to shot peening and observed a 33% increase in fatigue strength compared to untreated samples. The treatment also shifted crack initiation from the surface to the subsurface and reduced the influence of casting defects on fatigue cracking.

The authors of [8] emphasized the effectiveness of shot peening in enhancing the surface hardness of carbon steels. A series of experiments varying process parameters revealed a strong correlation between input conditions and resulting surface hardness. They concluded that appropriate selection and control of process parameters can help in achieving desired hardness levels. Koppula et al. [9] demonstrated that shot peening increased the surface hardness of a 2014 aluminum alloy from 115 HV to 131.5 HV. Similarly, the authors of [10] found that shot peening a Ti-6Al-4V alloy with zirconium ceramic balls produced a greater increase in microhardness than using steel balls, due to the unique combination of low density and high hardness of the ceramic shot. Moradi et al. [11] investigated the effect of shot peening time on various properties of a Ti-6Al-4V alloy. Hardness increased by 55%, 57%, and 63% for peening times of 20, 40, and 60 min, respectively. Residual surface stresses rose from 938 MPa after 20 min to 1232 MPa after 60 min. Microstructural analysis showed significant grain refinement, and surface roughness increased relative to the untreated material. According to [12], shot peening the 6082-T651 aluminum alloy at 8 bar working pressure increased surface roughness by more than 15 times. However, it also led to a 27% increase in microhardness and a deeper layer of residual compressive stresses. Ciuffini et al. [13] reported a reduction in surface roughness after shot peening AISI F55-UNS S32760 super duplex stainless steel. The treatment improved corrosion resistance, particularly against pitting, due to optimized microstructure and mechanical properties. Further, the authors of [14] noted a general enhancement in the stress corrosion cracking resistance of the Mg-8Gd-3Y alloy following shot peening.

The shot peening process can also significantly improve the tribological properties of materials. Wang et al. [15] observed a reduction in the volumetric wear of 42CrMoA steel compared to untreated samples. Additionally, a change in the wear mechanism was noted, from severe adhesive, fatigue, and abrasive wear to predominantly abrasive wear with only minor adhesive wear. Walczak et al. [16] also reported a positive effect of shot peening on tribological performance. In their study on DMLS 17-4PH steel in a 0.9% NaCl solution, both steel and ceramic shot were used, resulting in a significant reduction in the wear coefficient, by up to 32.7% in the most favorable variant. Interestingly, the friction coefficient increased by approximately 15–20% under the same conditions. The dominant wear mechanisms were abrasive and adhesive. Other researchers [17,18] also confirmed increased wear resistance after shot peening. The former conducted experiments on a Ti-6Al-4V alloy produced via DMLS (Direct Metal Laser Sintering) technology, while the latter investigated SAE 1070 steel. Similarly, Guan et al. [19], in their study on 25CrNi2MoV steel, observed a 45–65% reduction in wear volume and a decrease in friction coefficient values ranging from 15% to as much as 50%, depending on the shot intensity and size. Zhang et al. [20] reported improved tribological properties of 17Cr2Ni2MoVNb steel. In the most favorable processing variants, the friction coefficient was reduced by over 60%, and the wear coefficient by nearly 80%. The dominant wear mechanisms were identified as ploughing and abrasive wear.

In recent years, multi-criteria decision-making (MCDM) methods have attracted increasing interest. These approaches have proven to be highly effective tools for process optimization, informed decision-making, and enhancing product quality. Techniques such as TOPSIS and GRA have become particularly important across various fields of research. TOPSIS ranks alternatives by calculating their relative closeness to an ideal (best) and anti-ideal (worst) solution. It handles weighted criteria effectively, allowing decision-makers to prioritize outputs based on functional relevance. Its main advantages include intuitive interpretation, compatibility with normalized datasets, and wide adoption. However, it may be sensitive to outliers and depends on accurate normalization. In tribological research, Thirumalvalavan et al. [21] applied TOPSIS to optimize HVOF coating parameters for WC–Co nanocoating on a Ti-64 alloy, achieving a 42% reduction in wear and the friction coefficient under optimal conditions. GRA, in contrast, is suitable for systems with uncertain, incomplete, or small datasets. It transforms experimental values into a dimensionless form and evaluates closeness to an ideal reference using grey relational grades. GRA is computationally simple, less sensitive to data gaps, and provides robust performance in non-linear experimental systems. For example, Sylajakumari et al. [22] and Dharmalingam et al. [23] applied GRA to evaluate and optimize the wear behavior of metal–matrix composites under various test conditions. By combining both techniques, this study benefits from the quantitative precision and weighting structure of TOPSIS and the robust simplicity of GRA, allowing for complementary validation of results.

Alternative MCDM methods such as VIKOR, AHP, and MOORA are also used in studies related to tribological material selection and parameter optimization. Entropy–VIKOR has been applied to rank hybrid aluminum-based composites using sliding wear and friction data [24]. AHP combined with RSM was used to evaluate tribological performance (CoF, wear depth) of bionic-textured AISI 4140 composites in dry conditions [25]. And MOORA method has been used by researchers to evaluate and optimize wear and friction parameters in sliding tests of metal matrix composites, in a study involving red mud–reinforced aluminum composites [26]. Even though direct use of VIKOR, AHP, or MOORA for experimental sliding wear outputs is relatively rare, these methods are increasingly used for system-level decision-making in engineering applications. However, GRA and TOPSIS were selected in this study for their mathematical simplicity, low computational cost, robustness to data variation, and their complementary nature, which allows for verification of results through independent ranking logic.

In addition to experimental approaches, computational modeling has also become an essential tool for investigating tribological phenomena across multiple spatial and temporal scales. At the atomistic level, All-Atom Molecular Dynamics (MD) simulations provide detailed insights into the behavior of materials under sliding conditions, as demonstrated by Dašić et al. [27], in their study of trapped molecules at sliding contacts, where lattice-resolved friction was explored at the molecular scale. Reactive MD simulations enable the modeling of tribochemical processes by capturing dynamic bond formation and breakage, such as in the investigation of vanadium oxides under sliding conditions [28]. At the mesoscopic level, Coarse-Grained MD simulations are used to reduce computational complexity while retaining key physical interactions and have been effectively applied to study ionic liquid lubricants for automotive applications [29]. These simulation methods complement experimental findings by providing atomistic and mesoscale perspectives that help explain observed macroscopic behavior.

Although numerous studies investigate tribological behavior using factorial or Taguchi-based optimization, few works address the challenge of simultaneous multi-criteria evaluation (e.g., wear, friction, and weight loss) using experimental results. To fill this gap, the present study introduces a dual-MCDM approach combining TOPSIS and GRA to rank 27 test configurations of shot-peened 42CrMo4 steel based on tribological performance. The novelty lies in (1) the first-time application of these methods to this material and treatment combination, and (2) the comparative analysis of MCDM results to validate robustness in tribological optimization. This approach enables more informed decision-making for materials and process engineers facing competing performance metrics.

In this context, we hypothesize that applying Multi-Criteria Decision-Making (MCDM) techniques—specifically TOPSIS and GRA—can enhance the evaluation and optimization of tribological performance by providing an objective, data-driven ranking of experimental variants. These methods are especially useful when multiple conflicting tribological outputs must be considered simultaneously, allowing for a more comprehensive analysis than traditional single-output optimization.

## 2. Materials and Methods

**Figure 1:**
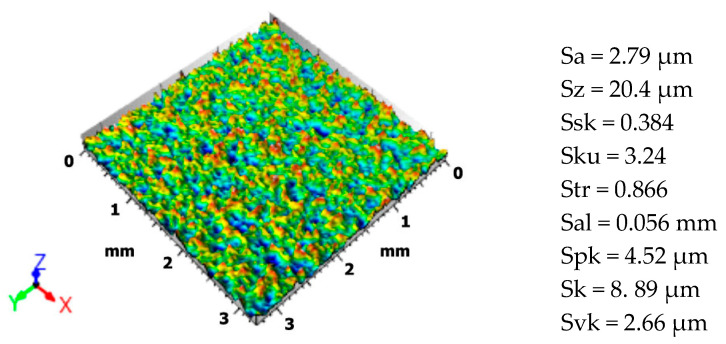
Isometric view of shot peened surface and selected surface topography parameters.

Tribological tests were conducted using the T-11 ball-on-disc tribological tester (manufactured by the Institute for Sustainable Technologies, the National Research Institute, Radom, Poland). The friction pair consisted of discs made from 42CrMo4 steel (hardness: 36 ± 2 HRC) and silicon carbide balls (hardness: 70 ± 2 HRC). The discs had a diameter of 25 mm and a height of 6 mm, while the balls were 6.35 mm in diameter. Tests were performed under dry friction conditions with rotary motion. A full-factorial L27 experimental design (each parameter combination tested) was used, varying three input parameters: load, sliding speed, and sliding distance. The specific levels of each parameter are presented in Table 1. The disc surfaces underwent a finishing treatment via shot peening, using spherical S-110 steel shots (hardness in the range of 45–52 HRC). Compared to other surface treatments such as coatings or laser surface texturing, shot peening offers several advantages. It enhances fatigue life, induces compressive residual stresses, and improves surface hardness without applying additional material layers, which can suffer from delamination. Moreover, shot peening is more cost-effective and industrially scalable, making it suitable for high-volume mechanical components. The peening was performed in a VaporBlast (Milwaukee, WI, USA) “VB 3576”, with a nozzle-to-surface distance of approximately 100 mm and an operating pressure of 0.6 MPa. The shot size was approximately 0.3 mm diameter (S-110 grade), and the exposure time was 4 min, achieving an estimated 100% coverage (i.e., a full overlapping impact across the surface). An isometric view of the disc surface, along with selected surface topography parameters [30,31], is shown in Figure 1. Since the shot peening parameters were identical for all samples, the surface topography image presented is representative of all specimens tested in this study. The parameters included are as follows: Sa, arithmetic mean surface height; Sz, maximum surface height; Ssk, surface skewness; Sku, surface kurtosis; Str, texture aspect ratio; Sal, autocorrelation length; Spk, reduced peak height; Sk, core roughness depth; and Svk, reduced valley depth. As a result of the shot peening process, the maximum microhardness of the treated specimens increased by approximately 15–20%, and the maximum compressive residual stresses achieved values between 660 and 680 MPa.

Prior to testing, the samples were weighed using a Radwag PS R1 laboratory scale. During the tests, the friction force was continuously recorded. Post-test, wear was assessed using a Talysurf CCI Lite (Taylor Hobson, Leicester, UK) white light interferometer. Each worn disc was measured in four evenly spaced positions (every 90 degrees), covering a surface area of 3.25 × 3.25 mm. In each area, eight profiles were extracted perpendicular to the wear track, and the cross-sectional wear area was calculated using TalyMap 6.2 Gold software [32]. These values were averaged and used to compute volumetric wear according to Equation (1). Additionally, discs were reweighed after testing, and weight loss was determined by subtracting the post-test weight from the initial value. The resulting tribological performance indicators were the following: volumetric wear, coefficient of friction, and weight loss.

(1) $$VD=\pi *d*S[{mm}^{3}]$$

where:

d is the diameter of the wear track [mm];

S is the cross-sectional area of wear [mm^2^].

To identify the most favorable test conditions, two multi-criteria decision analysis (MCDA) methods were applied: TOPSIS (Technique for Order Preference by Similarity to Ideal Solution) [33] and GRA (Grey Relational Analysis) [34]. Both methods are designed to evaluate and compare alternatives based on multiple criteria of varying importance and scale. Although their ultimate objective is the same—selecting the most advantageous variant—they rely on different mathematical principles and algorithms. Despite these methodological differences, both techniques are well-suited to tribological studies, where trade-offs often exist between wear, friction, and material stability. In such analyses, minimizing each individual parameter is important, but balancing them effectively is equally critical. Both MCDA methods are capable of capturing this balance, albeit in different ways.

The TOPSIS method [35] ranks alternatives based on their proximity to an ideal solution—defined by minimum friction coefficient, lowest volumetric wear, and smallest weight loss—and their distance from the worst-case (anti-ideal) solution. The step-by-step procedure of the TOPSIS method is as follows [33]:

1. Construction of the decision matrix.
2. Normalization of the decision matrix:

(2) $$r_{ij}=\frac{x_{ij}}{\sqrt{\sum_{i=1}^{m}x_{ij}^{2}}}$$

for *i* = 1, 2, …, *m* and *j* = 1, 2, …, *n*

3. Construction of the weighted normalized decision matrix:

(3) $$v_{ij}=w_{j}{*r}_{ij}$$

4. Determination of the ideal solution (A^+^) and the anti-ideal solution (A^−^):

(4) $$A^{+}=\left\{\left(\underset{i=1,\ldots ,m}{\max}v_{ij}|j\in J_{Q}\right),\left(\underset{i=1,\ldots ,m}{\min}v_{ij}|j\in J_{C}\right)\right\}$$

(5) $$A^{-}=\left\{\left(\underset{i=1,\ldots ,m}{\max}v_{ij}|j\in J_{Q}\right),\left(\underset{i=1,\ldots ,m}{\min}v_{ij}|j\in J_{C}\right)\right\}$$

where *J_Q_* is the set of stimulants and *J_C_* is the set of destimulants

Stimulants are criteria whose higher values are desirable (e.g., strength, efficiency), whereas destimulants are those for which lower values are preferred (e.g., wear, friction, weight loss).

5. Calculation of the Euclidean distance from the ideal and anti-ideal solutions:

(6) $$S_{i}^{+}=\sqrt{\sum_{j=1}^{n}{\left(v_{ij}-A_{j}^{+}\right)}^{2}}$$

(7) $$S_{i}^{-}=\sqrt{\sum_{j=1}^{n}{\left(v_{ij}-A_{j}^{-}\right)}^{2}}$$

for *i* = 1, 2, … and *m* and *j* = 1, 2, …, *n*.

6. Calculation of the closeness coefficient (C_i_), representing the relative proximity of each variant to the ideal solution:

(8) $$C_{i}=\frac{S_{i}^{-}}{S_{i}^{+}+S_{i}^{-}}$$

for *i* = 1, 2, … and *m* but $0\leq C_{i}\leq 1$.

Next, all variants are ranked in descending order based on their *C_i_* values. The variant with the highest *C_i_* is considered the best.

The Grey Relational Analysis (GRA) method is derived from grey system theory, which is particularly useful when the available information is incomplete or uncertain [34]. The steps are as follows:

1. Data normalization.

For “smaller-is-better” criteria (e.g., wear, friction, weight loss), normalization is performed using:

(9) $$x_{ij}^{\prime }=\frac{{max\left(x_{j}\right)-x}_{ij}}{max\left(x_{j}\right)-min\left(x_{j}\right)}$$

where

$x_{ij}$ is the original value of criterion *j* for variant *i*;

$x_{ij}^{\prime }$ is the normalized value;

$max\left(x_{j}\right),\;min\left(x_{j}\right)$ are the maximum and minimum values in column *j.*

2. Calculation of the deviation sequence.

The absolute difference between the normalized value and the reference (ideal) value is calculated.

(10) $${∆}_{ij}^{\prime }=\left|x_{0j}^{\prime }-x_{ij}^{\prime }\right|=\left|1-x_{ij}^{\prime }\right|$$

3. Calculation of the grey relational coefficient (GRC).

(11) $$\xi_{i}=\frac{{∆}_{min}+\zeta *{∆}_{max}}{{∆}_{ij}+\zeta *{∆}_{max}}$$

where

${∆}_{ij}$ is the deviation for the *i*th variant and *j*th criterion;

ζ is the distinguishing coefficient (typically 0.5);

${∆}_{min}$ and ${∆}_{max}$ are the minimum and maximum values among all deviations.

4. Calculation of the grey relational grade (GRG).

When weighting criteria, the GRG for each variant is calculated as follows:

(12) $$\gamma_{i}=\sum_{j=1}^{n}\omega_{j}*\xi_{ij}$$

where

$\gamma_{i}$ is the GRG for variant *i*;

$\omega_{j}$ is the weight for criterion *j*;

$\xi_{ij}$ is the grey relational coefficient for variant *i* and criterion *j*;

*n* is the number of criteria.

5. Ranking

Variants are ranked in descending order based on the value of $\gamma_{i}$. The highest GRG indicates the best-performing variant.

The analysis uses weighted values to reflect the practical importance of each tribological parameter. The highest weight (0.45) was assigned to the coefficient of friction (CoF), as it directly influences the operational stability and energy efficiency of the system. A weight of 0.35 was assigned to volumetric wear (VD), representing surface durability and wear resistance. The lowest weight (0.20) was assigned to weight loss (WL), which, although relevant for assessing material degradation, is generally of lesser practical significance compared to the other two criteria.

## 3. Results and Discussion

### 3.1. Surface Topography After the Shot Peening Process

**Figure 2:**
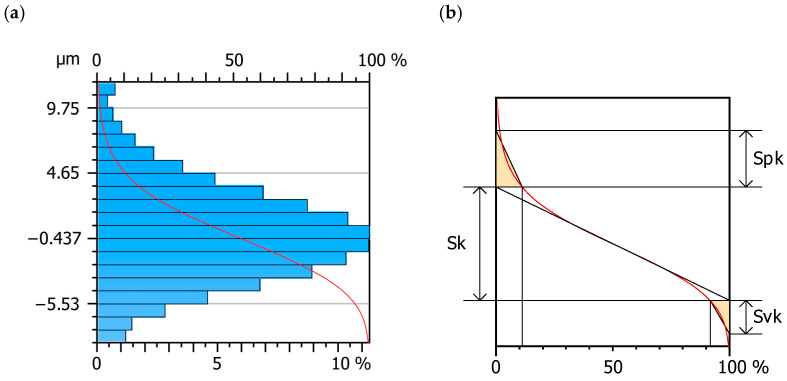
Surface height distribution histogram (**a**) and material ratio curve (**b**) with functional parameters (Spk, Sk, Svk). The red line represents the actual Abbott–Firestone curve (material ratio curve). The orange-colored areas indicate the reduced peak height (Spk) and the reduced valley depth (Svk), which are derived from deviations of the actual curve from the linear core roughness region (Sk).

The surface after the shot peening process exhibited a clearly developed texture dominated by small micro-convexities. The arithmetic mean surface height (Sa) was 2.83 µm. The positive skewness value (Ssk = 0.533) indicates a predominance of peaks over valleys, suggesting that convexities created by ball impacts dominate the surface morphology. These convex features can be rapidly flattened during the initial stages of operation, contributing to the stabilization of contact conditions. This asymmetry is also reflected in the distribution of surface height values (Figure 2a), which is largely influenced by the geometry of the surface and the variation in material-to-void ratio as a function of depth.

In this case, the surface profile closely resembles a normal distribution, with the highest surface load-bearing capacity occurring at a depth of –0.437 µm. Analysis of the Abbott–Firestone load-bearing curve (Figure 2b) revealed a relatively high reduced peak height (Spk = 4.52 µm) and a lower reduced valley depth (Svk = 2.66 µm). This suggests that the surface contains numerous ridges that are easily worn down during service. The core roughness depth (Sk = 8.89 µm) indicates good load-bearing capacity, which, once the peaks are flattened, contributes to enhanced contact stability. While Svk is lower than Spk, it is still significantly higher than values typically observed in conventional finishing processes—which usually range from 0.1 to 1.0 µm depending on the method used—implying the presence of valleys capable of retaining lubricant. This is further supported by the volumetric parameters: the core material volume (Vmc) was 3.22 mL/m^2^, and the core void volume (Vvc) was 4.64 mL/m^2^, confirming the surface’s ability to collect and retain lubricant during operation.

**Figure 3:**
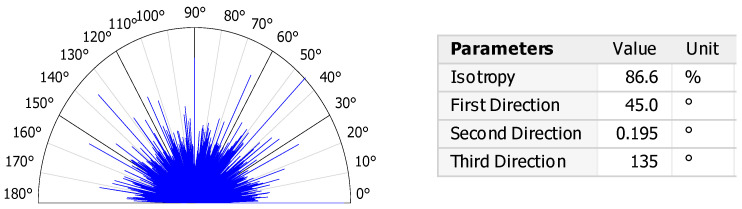
Directionality of the surface texture.

The arrangement of surface features—whether ordered or random—is described by the degree of isotropy of the structure [36]. Surface isotropy refers to a uniform texture in all directions, corresponding to an ideally symmetrical structure relative to any axis of symmetry [37]. Conventionally, surfaces with an isotropy degree below 20% are considered anisotropic, while those above 80% are considered isotropic. As shown in Figure 3, the shot-peened surface demonstrated an isotropy level of 86.6%, which is typical of an isotropic structure.

**Figure 4:**
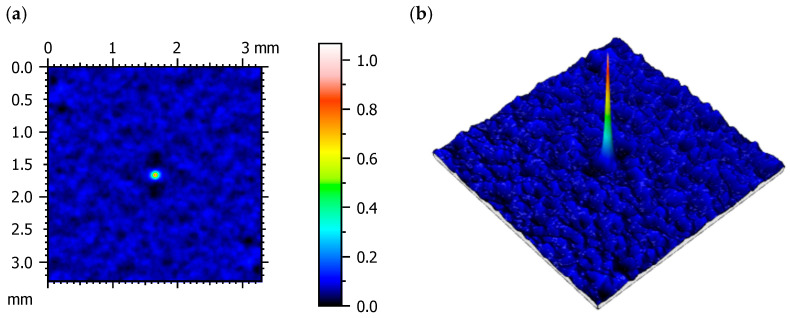
Autocorrelation plot of the surface texture: map of the surface (**a**) and isometric view (**b**).

Autocorrelation function analysis (Figure 4) confirmed the surface’s isotropic and random nature, with no preferred direction of feature orientation. The central correlation peak diminished rapidly, indicating a lack of long-range periodicity. This random, non-directional texture is advantageous in applications involving variable load directions, as it reduces the risk of fatigue cracking and directional wear marks [38].

**Figure 5:**
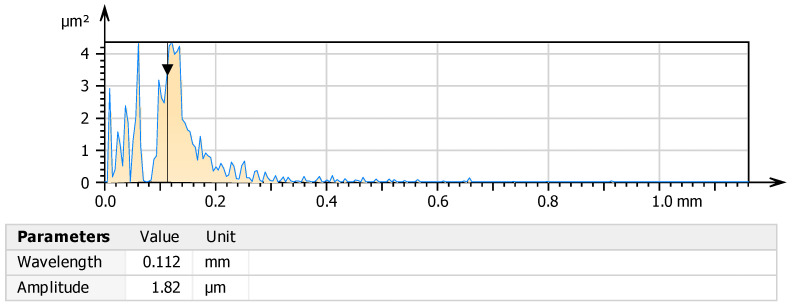
Power spectral density (PSD) chart after the shot peening process. The black arrow marks the main peak of the PSD, corresponding to the dominant surface wavelength (0.112 mm) and its associated amplitude (1.82 μm).

The power spectral density (PSD) function (Figure 5), which is the Fourier transform of the autocorrelation function, describes how surface irregularities are distributed over spatial frequencies [39]. In surface analysis, it is assumed that machining marks are perpendicular to the direction indicated by the PSD. For the shot-peened surface, the dominant surface wavelength was 0.112 mm, with a corresponding structure amplitude of 1.82 µm. This indicates a surface composed of uniform, fine topographic features with high spatial frequency, which is characteristic of this treatment method. Additionally, the autocorrelation length (Sal) was 0.056 mm, confirming a very short-range similarity among surface features and further indicating a highly irregular and fine-grained structure [40].

### 3.2. Tribological Properties

**Figure 6:**
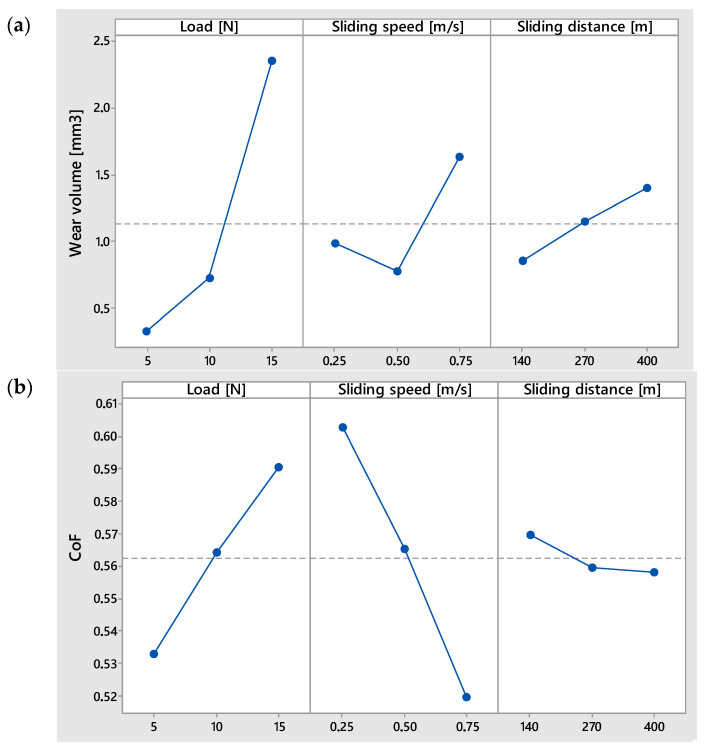

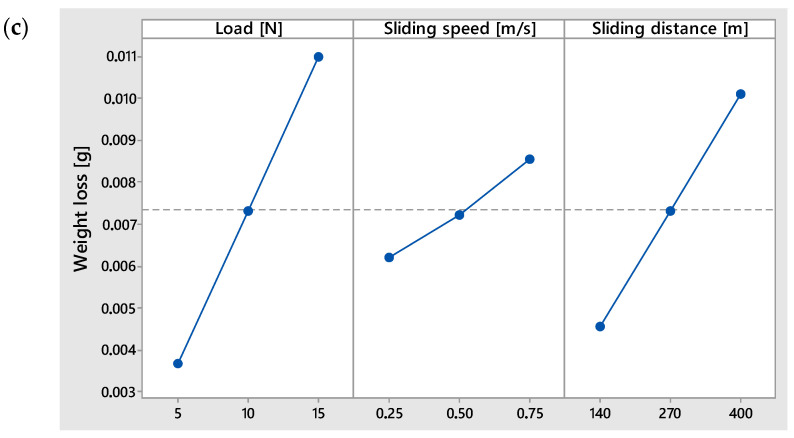
Influence of input parameters on wear volume (**a**), coefficient of friction (**b**), and weight loss (**c**).

The results showing the influence of input parameters on the tribological properties of the surface after the shot peening process are presented in Table 2 and Figure 6. Among the analyzed parameters, load exerted the most dominant influence on volumetric wear, which increased significantly from approximately 0.5 mm^3^ at a load of 5 N to over 2.3 mm^3^ at 15 N. This trend is consistent with the Archard wear law, which states that volumetric wear is proportional to the normal load and sliding distance under constant hardness conditions [41]. This trend is attributed to the rise in unit pressure, which leads to more intense mechanical removal of material. Such a relationship is typical and results from increased abrasive or adhesive interactions under higher contact pressures [42].

The effect of sliding distance was also noticeable, although less steep. Volumetric wear rose from <1 mm^3^ at 140 m to >1.5 mm^3^ at 400 m. This can be attributed to cumulative abrasion over time, but the non-linear nature of this trend suggests a running-in period, during which initial asperities are removed and real contact area stabilizes, which is a behavior typical for metallic friction pairs [43]. In contrast, the influence of sliding speed on volumetric wear was non-linear and ambiguous. The lowest wear volume was recorded at 0.5 m/s, slightly higher at 0.25 m/s, and the highest at 0.75 m/s. The increased wear at 0.75 m/s may be due to higher frictional energy and the resulting intensification of wear processes, as elevated speeds can increase interface temperature, reduce material hardness, and enhance surface fatigue [44].

As shown in Figure 6b, increasing the load from 5 N to 15 N also led to a rise in the coefficient of friction (CoF), from approximately 0.53 to over 0.59. This observation aligns with findings by other researchers [45], who explain the phenomenon as a result of increased real contact area and stronger adhesive forces under higher loads [46,47]. Formation of micro-welds and their cracking during sliding may further raise the CoF in metallic systems [43]. The relationship between sliding distance and CoF was relatively weak, though a slight decreasing trend was noted from about 0.57 at shorter paths to approximately 0.555 at 400 m. This behavior may be explained by contact conditioning and surface smoothing, reducing friction fluctuations after the initial sliding phase. Such stabilization effects have been linked to the transition from boundary to mixed lubrication regimes in similar systems [48]. In contrast, sliding speed demonstrated an inverse correlation with CoF. The highest friction coefficient was observed at 0.25 m/s (~0.59), while the lowest occurred at 0.75 m/s (~0.51). This trend may reflect a shift in dominant friction mechanism. At higher speeds, local surface temperatures can trigger the formation of oxide films, which act as solid lubricants and lower the shear strength at the interface [42,49]. Such decoupling of friction and wear behavior has been documented in steel pairs: Okonkwo et al. [50] observed adhesive-to-oxide-wear transition with increasing speed, whereas Sun et al. [51] reported decreasing CoF alongside increasing oxidative wear in 18Ni(300) maraging steel at high speeds.

Among all input parameters, load had the strongest effect on mass loss (Figure 6c). Weight loss increased from an average of 0.003 g at 5 N to 0.012 g at 15 N, corresponding to higher contact forces and intensified abrasion. This relationship between contact force and mechanical degradation aligns well with Hutchings’ research findings [52]. The friction path also significantly affected mass loss: the highest losses were observed for the longest path (400 m), while the shortest path resulted in the least wear, likely due to cumulative material removal over time. Interestingly, sliding speed had a moderate effect, with only slight increases in wear at higher velocities. This suggests a threshold effect, beyond which thermal contributions to wear become significant.

### 3.3. TOPSIS Analysis

The normalized input data were converted into a dimensionless form using vector normalization and weighted normalization methods. The weights assigned to the individual criteria were as follows: volumetric wear (VD) = 0.35, coefficient of friction (CoF) = 0.45, and weight loss (WL) = 0.2. The selection of the weights was based on the relative functional importance of each criterion in typical tribological applications, supplemented by references to a few studies using similar methods [53,54]. The coefficient of friction (CoF) was given the highest weight (0.45), reflecting its direct impact on energy efficiency and operating stability. Volumetric wear (VD) was assigned a slightly lower weight (0.35), as it affects durability and service life, while weight loss (WL) was considered the least critical in this context and given a weight of 0.2. This weighting scheme follows a common practice in multi-criteria decision-making, where expert judgment or engineering relevance guides weight assignment when no universal standard exists.

Based on the calculated distances from the ideal solution (*A^+^*) and the anti-ideal solution (*A^−^*), the closeness coefficient Ci was determined for each variant, forming the basis for ranking. The calculation results are presented in Table 3.

The *Ci* values ranged from 0.0782 to 0.9535, indicating a significant spread in the overall efficiency of the tested configurations. This range highlights a diverse spectrum of tribological behaviors among the variants. A similar approach to the evaluation of TOPSIS methods was used to assess the tribological characteristics of aluminum composites in the study by the authors of [53], where the minimization of CoF and wear was the objective of a multi-objective optimization using TOPSIS combined with GRA.

The best variants (Rank 1–10, *Ci* > 0.86) were characterized by a favorable balance between all three criteria, particularly a very low coefficient of friction, which had the greatest influence due to its higher weight. Despite some of them showing moderate wear values, the low CoF and WL ensured their high overall ranking. The best-performing variant (Rank 1, *Ci* = 0.9535) exhibited an excellent compromise between minimized wear and friction, indicating near-ideal tribological behavior. Mid-ranked variants (Rank 11–20, *Ci* ≈ 0.65–0.86) demonstrated a compromise in one of the output parameters, typically either slightly elevated volumetric wear or mass loss. While still acceptable, these configurations may require adjustment depending on specific application priorities. Notably, some variants with good wear resistance but relatively higher CoF values ranked lower, confirming the dominant influence of friction in the decision-making process. The remaining variants recorded unfavorable values in at least two of the three criteria, most frequently very high volumetric wear (VD > 2.0 mm^3^) and noticeable weight loss (WL > 0.010 g). The use of differentiated weights allowed for a more realistic reflection of the importance of each criterion. The high weight of the CoF (0.45) strongly impacted the overall rankings, differentiating variants with similar wear levels but different frictional characteristics.

**Figure 7:**
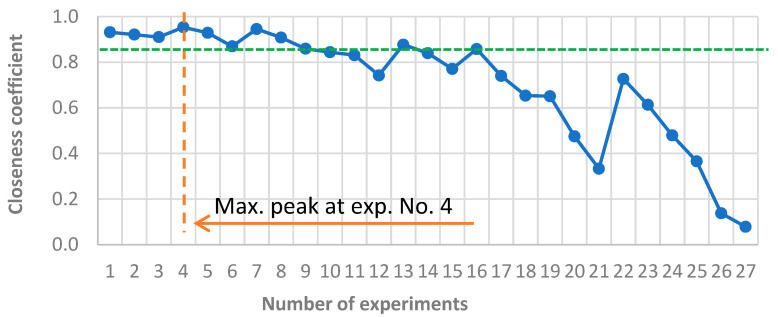
Ranking chart of the results obtained in the TOPSIS method. The blue line represents the closeness coefficient calculated for each of the 27 experiments. The orange dashed line and arrow highlight the experiment with the maximum closeness value (No. 4), and the green dashed line indicates a reference threshold.

For the purpose of graphical presentation, a ranking chart was created (Figure 7), on which an effectiveness threshold was identified. The graph shows the effectiveness threshold, which was assumed at a closeness coefficient level of 0.86 (green line). Ten variants (No. 1–9 and 13) exceeded this value and can therefore be considered optimal or strongly preferred in the analyzed system.

The results indicate that the TOPSIS method enables not only the identification of the best-performing variant but also the classification of all experimental variants according to the degree of compromise among the criteria.

### 3.4. GRA Method

The Grey Relational Analysis (GRA) method was applied as a complementary multi-criteria decision-making tool. The Grey Relational Grade (GRG) values ranged from 0.3907 to 0.8675 (Table 4), with the top configurations again corresponding to low CoF and moderate wear/mass loss. This strong agreement with the TOPSIS results reinforces the robustness of the experimental evaluation.

The highest GRG score (0.8675) was achieved by a variant exhibiting both minimal CoF (≈0.51) and low to moderate wear levels, confirming the dominant influence of friction on performance ranking. This finding echoes earlier reports that friction reduction often leads to significantly improved system durability, even when minor increases in wear are observed [55].

Based on the resulting rankings, the following observations can be made:

- The top-performing variants (GRG > 0.7) exhibited a low volumetric wear, low friction coefficient, and moderate mass loss. These configurations represent potentially optimal settings of input parameters for tribological testing.
- Middle-ranked variants (GRG between 0.5 and 0.7) demonstrated certain trade-offs; typically, at least one output parameter remained significantly less favorable, which lowered the overall efficiency rating.
- The lowest-ranked variants (GRG < 0.5) were associated with at least two unfavorable parameters—most often very high wear (VD > 2 mm^3^) and significant mass loss (>0.010 g)—which significantly penalized their final scores, particularly due to the weighted evaluation system.

**Figure 8:**
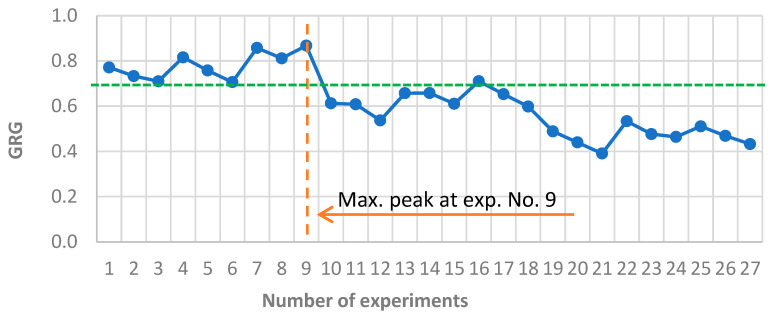
Ranking chart of the results obtained in the GRA method. The blue line represents the GRG coefficient calculated for each of the 27 experiments. The orange dashed line and arrow highlight the experiment with the maximum GRG value (No. 9), and the green dashed line indicates a reference threshold.

Similarly to the TOPSIS method, a ranking chart (Figure 8) of the obtained results was prepared, including an efficiency threshold set at 0.7.

The GRA results also confirm the importance of appropriate weight assignment, particularly for CoF, which, when given high priority, helped differentiate closely performing variants. For example, variants with moderate VD but high CoF received lower GRG scores than those with slightly higher wear but more favorable friction values. As a result, the GRA method effectively incorporated both the individual and collective importance of each parameter, yielding a clear and well-justified efficiency ranking.

From a practical standpoint, the GRA analysis can provide engineers with quite a good tool for identifying the most sustainable test variants, taking into account application-specific priorities (e.g., minimizing friction at the expense of acceptable wear). The generated ranking can serve not only in designing optimal operating conditions for friction pairs but also as a valuable starting point for further material development and simulation studies.

### 3.5. TOPSIS vs. GRA Comparison

The comparative analysis of the rankings obtained via TOPSIS and GRA revealed a high degree of consistency, especially among top-performing variants. For instance:

- Variant No. 4 was ranked 1st in TOPSIS and 3rd in GRA;
- Variant No. 1 appeared in the TOP 5 for both methods;
- Variant No. 7 was ranked 2nd in both TOPSIS and GRA;
- Variants No. 2, 5, and 8 also consistently appeared in the top 7, confirming the reliability of the rankings.

Such convergence is particularly valuable in multi-criteria decision-making, as it increases confidence in the robustness of the identified optimal solutions. However, moderate deviations were observed in the middle-ranked configurations (Ranks 10–20). These discrepancies likely stem from differences in algorithmic logic: the TOPSIS method, which is based on the analysis of geometric distances from the ideal and anti-ideal solutions, is more sensitive to extreme values of individual criteria. As a result, some variants that achieved high GRG values were ranked slightly lower by TOPSIS due to a larger deviation from the ideal in one of the criteria.

To quantitatively assess the agreement between the rankings obtained by the TOPSIS and GRA methods, the Spearman’s rank correlation coefficient (ρ) was calculated. The result was ρ = 0.934, indicating a very strong positive correlation between the two rankings. This confirms that both methods generally identify similar performance trends.

Although both TOPSIS and GRA were applied using the same input criteria and the same weighting system (with CoF assigned the highest importance of 0.45), some variation in the rankings was observed. These differences are not due to inconsistencies in preference but rather stem from methodological differences: TOPSIS emphasizes geometric distance from ideal and anti-ideal solutions, while GRA focuses on normalized relational closeness. As a result, certain mid-ranked configurations—especially those with strong performance in one criterion but moderate performance in others—may be scored differently, even though the same priorities were applied.

**Figure 9:**
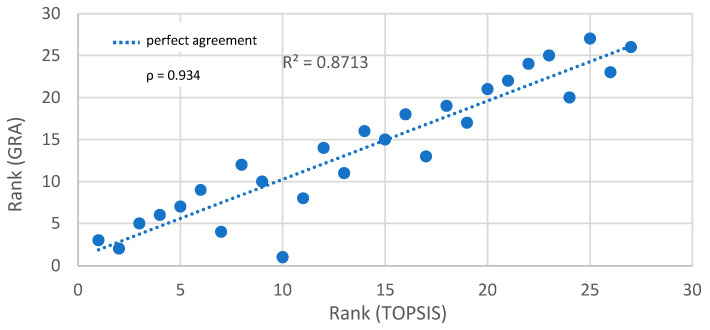
Comparison of ranks obtained by TOPSIS and GRA methods.

The comparison plot, including the correlation coefficient, is shown in Figure 9. The dashed blue line represents perfect agreement (identity line). The Spearman’s rank correlation coefficient between the two rankings is ρ = 0.934, indicating very strong agreement.

The use of both methods in parallel provides a complementary decision-support framework. TOPSIS is particularly well-suited for scenarios requiring custom prioritization of criteria, as in industrial tribological systems where friction may be critical for energy efficiency or component life. Therefore, it is recommended that TOPSIS be used as the primary optimization tool in cases where engineering priorities are clearly defined, while GRA can serve as a valuable cross-check to ensure that selected configurations are not overly biased by a single parameter and remains effective even in exploratory scenarios where decision-maker preferences may still be evolving or debated.

### 3.6. Weight Sensitivity Analysis

To evaluate the robustness of the obtained rankings with respect to changes in weighting preferences, a weight sensitivity analysis was conducted for both the TOPSIS and GRA methods. The original weight configuration prioritized the coefficient of friction (CoF = 0.45), followed by volumetric wear (VD = 0.35), and weight loss (WL = 0.20). In this analysis, we examined how the rankings changed under three alternative scenarios:

- Scenario A (Friction-prioritized): CoF = 0.5, VD = 0.3, WL = 0.2;
- Scenario B (Balanced weights): CoF = 0.33, VD = 0.33, WL = 0.34;
- Scenario C (Wear-prioritized): CoF = 0.3, VD = 0.5, WL = 0.2.

The methods were reapplied using each of these configurations, and rankings were compared to the original outcome. The top-performing alternatives—particularly variants 4, 1, and 7—remained within the top five in all scenarios. This consistency confirms the robustness and reliability of the optimization results across varying application-specific priorities. Slight shifts in mid-ranked variants were observed, which reflect the expected behavior of MCDM methods when prioritization changes. Nevertheless, no significant reordering occurred in the top or bottom ranks.

**Figure 10:**
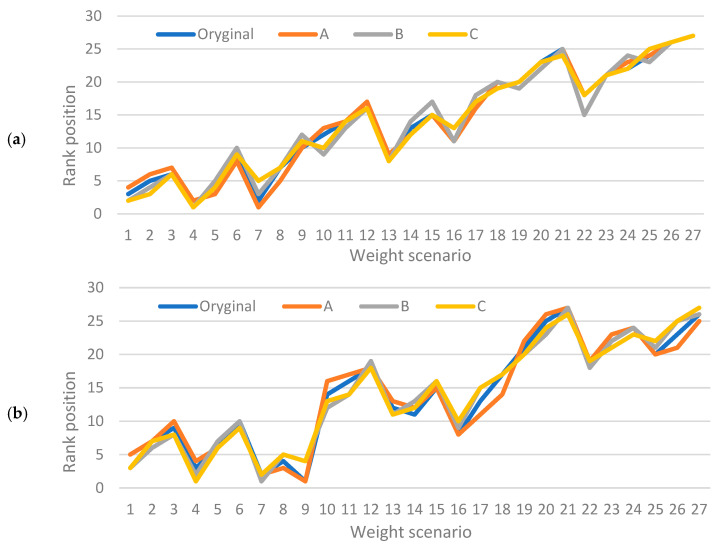
Stability of ranking positions across all weight configurations for the TOPSIS (**a**) and GRA (**b**) methods.

Figure 10 illustrates the stability of the ranking positions across all weight configurations for the TOPSIS (a) and GRA (b) methods.

The generated rankings can serve not only in designing optimal operating conditions for friction pairs but also as a valuable starting point for further material development and simulation studies. In particular, the identified optimal and sub-optimal tribological configurations may inform the boundary conditions and validation datasets for multiscale simulation approaches. At the atomistic level, All-Atom and Reactive Molecular Dynamics (MD) simulations can be employed to investigate fundamental mechanisms of wear, friction, and tribochemical reactions under similar contact loads and sliding velocities [27,28]. At the macro-scale, models based on Finite Element Method (FEM), Finite Volume Method (FVM), or Elastohydrodynamic Lubrication (EHL) theories can simulate stress distribution, heat generation, and lubrication conditions for real-world friction contacts [42]. Integrating experimental MCDM-based optimization with simulation frameworks offers a pathway toward physically informed materials design and predictive tribological modeling, especially when extended by data-driven or AI-based approaches in future studies.

## 4. Conclusions

The main conclusions drawn from the experimental results and multi-criteria analysis are summarized below:

✓ The shot peening process significantly modified the surface topography, increasing surface isotropy and enhancing load-bearing properties.
✓ Tribological tests showed that load had the strongest influence on all output parameters (wear volume, coefficient of friction, and weight loss), followed by sliding distance and speed.
✓ The application of multi-criteria decision-making (MCDM) methods—TOPSIS and GRA—enabled a comprehensive optimization of test conditions based on three conflicting output parameters. Both the TOPSIS and GRA methods provided consistent rankings, with Spearman’s rank correlation coefficient reaching 0.934, confirming the reliability of the selected solutions.
✓ The best-ranked configurations, particularly variants No. 4, 1, and 5, which met all predefined threshold criteria (VD < 0.22 mm^3^; CoF < 0.57; WL < 0.005 g), are recommended as optimal. These limit values were selected based on percentile analysis of the results (approx. lower quartile), technical relevance to tribological systems, and engineering judgement to ensure a balance between friction reduction and wear resistance. Variant No. 7 also ranked highly in both methods and can be considered a near-optimal solution.
✓ A weight sensitivity analysis confirmed the robustness of the top-performing variants across three different weighting scenarios.
✓ GRA proved to be less sensitive to individual outliers, making it a valuable complementary tool to distance-based methods like TOPSIS.

From a methodological perspective, TOPSIS offered greater control over the decision-making process, particularly through its ability to reflect trade-offs between conflicting criteria via weighting. GRA, on the other hand, demonstrated stability and simplicity, offering valuable validation of the ranking results and minimizing the effect of extreme deviations in individual tribological parameters (e.g., wear volume or CoF), which may distort rankings in other methods.

## 5. Limitations and Future Work

✓ The present study was limited to dry sliding conditions using a specific ball-on-disc configuration. The influence of lubrication, temperature variation, and environmental exposure (e.g., humidity, corrosion) was not considered.
✓ Only one material pair (42CrMo4 steel vs. SiC) was investigated. Additional studies should include alternative substrate materials and counter-body types to generalize the findings.
✓ The tribological behavior was assessed at macro scale. Micro- or nano-scale investigations could offer more detailed insights into the mechanisms of wear and friction.
✓ The current weighting scheme in MCDM was based on assumed engineering priorities. Future work could integrate stakeholder-based preference modeling or machine learning techniques to dynamically adjust weights.
✓ Future research should also explore the hybridization of optimization techniques (e.g., combining GRA or TOPSIS with genetic algorithms), as well as the use of AI-assisted modeling to predict tribological outcomes under a wide range of conditions.

## Figures and Tables

**Table 1:** Levels of tribological parameters.

Levels	Load [N]	Sliding Speed [m/s]	Sliding Distance [m]
1	5	0.25	140
2	10	0.50	270
3	15	0.75	400

**Table 2:** Results of the experimental investigations.

No.	Input Parameters			Output Variables		
	P [N]	v [m/s]	s [m]	VD [mm^3^]	CoF	WL [g]
1	5	0.25	140	0.169	0.565	0.002
2	5	0.25	270	0.201	0.572	0.003
3	5	0.25	400	0.233	0.571	0.004
4	5	0.5	140	0.174	0.536	0.002
5	5	0.5	270	0.212	0.541	0.004
6	5	0.5	400	0.571	0.532	0.005
7	5	0.75	140	0.357	0.499	0.003
8	5	0.75	270	0.477	0.502	0.004
9	5	0.75	400	0.584	0.479	0.006
10	10	0.25	140	0.568	0.622	0.004
11	10	0.25	270	0.607	0.589	0.006
12	10	0.25	400	0.868	0.606	0.009
13	10	0.5	140	0.474	0.585	0.004
14	10	0.5	270	0.531	0.549	0.007
15	10	0.5	400	0.653	0.555	0.01
16	10	0.75	140	0.642	0.526	0.005
17	10	0.75	270	0.972	0.519	0.009
18	10	0.75	400	1.216	0.526	0.012
19	15	0.25	140	1.459	0.645	0.006
20	15	0.25	270	2.168	0.621	0.009
21	15	0.25	400	2.635	0.633	0.013
22	15	0.5	140	1.088	0.606	0.007
23	15	0.5	270	1.429	0.609	0.011
24	15	0.5	400	1.914	0.575	0.015
25	15	0.75	140	2.783	0.544	0.008
26	15	0.75	270	3.762	0.536	0.013
27	15	0.75	400	3.957	0.546	0.017

**Table 3:** TOPSIS results.

No	Weighted Normalized Value			Separation Values		*Ci* Value	Rank
	VD [mm^3^]	CoF	WL [g]	*A^+^*	*A^−^*		
1	0.0074	0.0867	0.0092	0.0132	0.1792	0.9314	3
2	0.0088	0.0878	0.0138	0.0151	0.1761	0.9212	5
3	0.0101	0.0877	0.0184	0.0171	0.1732	0.9102	6
4	0.0076	0.0823	0.0092	0.0088	0.1794	0.9535	1
5	0.0092	0.0830	0.0184	0.0134	0.1744	0.9288	4
6	0.0249	0.0817	0.0230	0.0237	0.1584	0.8697	9
7	0.0155	0.0766	0.0138	0.0099	0.1709	0.9454	2
8	0.0208	0.0771	0.0184	0.0166	0.1643	0.9080	7
9	0.0254	0.0735	0.0276	0.0258	0.1574	0.8602	10
10	0.0247	0.0955	0.0184	0.0295	0.1593	0.8439	12
11	0.0264	0.0904	0.0276	0.0314	0.1546	0.8311	14
12	0.0378	0.0930	0.0414	0.0484	0.1395	0.7424	16
13	0.0206	0.0898	0.0184	0.0229	0.1633	0.8769	8
14	0.0231	0.0843	0.0322	0.0299	0.1568	0.8399	13
15	0.0284	0.0852	0.0461	0.0440	0.1480	0.7708	15
16	0.0279	0.0807	0.0230	0.0258	0.1556	0.8577	11
17	0.0423	0.0797	0.0414	0.0479	0.1364	0.7400	17
18	0.0529	0.0807	0.0553	0.0652	0.1229	0.6535	19
19	0.0635	0.0990	0.0276	0.0643	0.1200	0.6509	20
20	0.0944	0.0953	0.0414	0.0953	0.0862	0.4750	23
21	0.1147	0.0972	0.0599	0.1210	0.0604	0.3331	25
22	0.0473	0.0930	0.0322	0.0501	0.1333	0.7268	18
23	0.0622	0.0935	0.0507	0.0716	0.1136	0.6134	21
24	0.0833	0.0883	0.0691	0.0978	0.0900	0.4792	22
25	0.1211	0.0835	0.0368	0.1175	0.0676	0.3653	24
26	0.1638	0.0823	0.0599	0.1646	0.0263	0.1377	26
27	0.1722	0.0838	0.0783	0.1791	0.0152	0.0782	27

**Table 4:** GRA results.

No	Normalized Values			GRC			GRG Grade	Rank
	VD [mm^3^]	CoF	WL [g]	VD [mm^3^]	CoF	WL [g]		
1	1.0000	0.4819	1.0000	1.0000	0.4911	1.0000	0.7710	5
2	0.9916	0.4398	0.9333	0.9835	0.4716	0.8824	0.7329	7
3	0.9832	0.4458	0.8667	0.9675	0.4743	0.7895	0.7100	9
4	0.9988	0.6566	1.0000	0.9976	0.5929	1.0000	0.8159	3
5	0.9887	0.6265	0.8667	0.9778	0.5724	0.7895	0.7577	6
6	0.8939	0.6807	0.8000	0.8249	0.6103	0.7143	0.7062	10
7	0.9504	0.8795	0.9333	0.9098	0.8058	0.8824	0.8575	2
8	0.9187	0.8614	0.8667	0.8601	0.7830	0.7895	0.8113	4
9	0.8904	1.0000	0.7333	0.8203	1.0000	0.6522	0.8675	1
10	0.8948	0.1386	0.8667	0.8262	0.3673	0.7895	0.6123	14
11	0.8845	0.3373	0.7333	0.8123	0.4301	0.6522	0.6083	16
12	0.8155	0.2349	0.5333	0.7305	0.3952	0.5172	0.5370	18
13	0.9197	0.3614	0.8667	0.8616	0.4392	0.7895	0.6571	12
14	0.9045	0.5783	0.6667	0.8397	0.5425	0.6000	0.6580	11
15	0.8723	0.5422	0.4667	0.7965	0.5220	0.4839	0.6105	15
16	0.8752	0.7169	0.8000	0.8003	0.6385	0.7143	0.7103	8
17	0.7881	0.7590	0.5333	0.7024	0.6748	0.5172	0.6529	13
18	0.7238	0.7169	0.3333	0.6441	0.6385	0.4286	0.5985	17
19	0.6596	0.0000	0.7333	0.5949	0.3333	0.6522	0.4887	21
20	0.4724	0.1446	0.5333	0.4866	0.3689	0.5172	0.4397	25
21	0.3490	0.0723	0.2667	0.4344	0.3502	0.4054	0.3907	27
22	0.7576	0.2349	0.6667	0.6734	0.3952	0.6000	0.5336	19
23	0.6674	0.2169	0.4000	0.6005	0.3897	0.4545	0.4764	22
24	0.5393	0.4217	0.1333	0.5204	0.4637	0.3659	0.4640	24
25	0.3101	0.6084	0.6000	0.4202	0.5608	0.5556	0.5105	20
26	0.0514	0.6566	0.2667	0.3452	0.5929	0.4054	0.4687	23
27	0.0000	0.5964	0.0000	0.3333	0.5533	0.3333	0.4323	26

## Data Availability

The original contributions presented in this study are included in the article. Further inquiries can be directed to the corresponding author.
